# The Child’s Right to Know Versus the Parents’ Right Not to Tell: The Attitudes of Couples Undergoing Fertility Treatments Towards Identity-Release Gamete Donation

**DOI:** 10.18502/jri.v24i3.13276

**Published:** 2023

**Authors:** Douglas Oliveira Rocha, Rose Marie Massaro Melamed, Daniela Paes de Almeida Ferreira Braga, Amanada Souza Setti, Assumpto Iaconelli, Edson Borges

**Affiliations:** 1- Associação Instituto Sapientiae-Centro de Estudos e Pesquisa em Reprodução Assistida, São Paulo, Brazil; 2- Fertility Medical Group, São Paulo, Brazil

**Keywords:** Donor conception, Donor, Oocyte donation, Sperm, Survey

## Abstract

**Background:**

In Brazil, donor anonymity is mandatory; however, the tendency of Brazilians towards the practice is unknown. In this study, an attempt was made to investigate whether couples undergoing assisted reproductive technology (ART) have a different perception of anonymous versus identity-release gamete donation than a target population in Brazil.

**Methods:**

This cross-sectional study was performed from September 1, 2020 to December 15, 2020. For that purpose, surveys through online platforms were conducted, including either patients undergoing ART (ART-group, n=400) or subjects interested in the theme (interested-group, n=100) randomized by age at a 1:4 ratio. The survey collected information on the participants’ attitudes towards anonymity of gamete donors, and answers were compared between the groups.

**Results:**

Most participants stated that the relationship between children and their parents would be affected by the child’s knowledge of the origin of its conception. Most participants in the ART-group believed that the gamete donor’s identity should not be revealed to the child, while only half of the interested-group stated the same. Most of the participants stated that “the donor’s identity should be revealed if the child questions its biological origin”. “From birth” was the second most common response, while “when the child turns 18 years old” and “sometime during teenage years” were less common answers.

**Conclusion:**

The attitudes of ART patients about anonymity are conservative, with most participants believing that family relationships may be affected if the child is aware of the origin of his/her conception. These patients also believe that the identity of the gamete donor should not be revealed to the child.

## Introduction

Gamete donation is a well-established treatment in ART (1). Sperm donation has been used for more than a century (2) and is cur rently used for male factor infertility, paternally inherited genetic disorders, or absence of a male partner (3). The first practice of oocyte donation was done 30 years ago (4) and is currently applied in cases of maternally inherited genetic disorders and premature ovarian failure, repeated implantation failure, menopausal women, poor-responders, and absence of a female partner (5).

Over the past years, fertility treatments using donated oocytes have widely increased, and not only are a large number of children born following donor conception (5, 6), but there is also an increasing demand for donor conception worldwide. An important question in gamete donation is whether donor-conceived children should be informed about the facts of their conception and, if so, how much information about the donors should be revealed.

Donor conception has traditionally been performed with anonymous donors (7); however, lately, there is a global trend towards programs using donors that are identifiable to the resulting offspring. The use of identity-release donors implies that the donor is anonymous to the recipients, although they may receive some non-identifying information about the donor. Upon request from a donor-conceived child who has reached mature age, the donor’s identity is released to the child (7).

Currently, legislation concerning whether donor conception should be anonymous ranges from mandating donor anonymity in some countries, such as Brazil, to prohibition of anonymous gamete donation in others. In some countries, such as Japan, there is no statutory law regulating ART or third-party reproduction (8). In one way or another, ART legislation and recommendations concerning third-party reproduction are increasingly promoting openness to the child about the origin of the gametes (9).

Legislation on identity-release gamete donation was first introduced in 1985 in Sweden (10), and currently, it is mandatory in several countries, including Finland, Sweden, the Netherlands, and the United Kington (11). In the United States, legal requirements concerning recordkeeping and releasing donor information vary from state to state (12). Open-identity donors in the United States typically provide extensive non-identifying information for recipients and, when the offspring reaches the age of 18 years, provide their name and sometimes other identifying information to offspring who requests it (13).

In Brazil, the last resolution of the National Health Surveillance Agency (ANVISA) published in December 2022, determined that confidentiality of all information relating to the sample to be used must be respected. The assisted reproduction center is responsible to preserve the confidentiality of documents and records involving gamete donors (14).

Since the 1990s, a growing debate about whether donor-conceived people should have a legal right to access information about their donor has been observed; yet a shift in attitudes towards a more open view has emerged which argues that donor-conceived people should be told about the way they were conceived and they must have access to information about their gamete donor (15–18).

It has been argued that identity-release gamete donation protects the offspring’s interest in knowing their genetic heritage, securing accurate information about potential health problems, and making future medical decisions. In contrast, those who defend anonymous gamete donation state that donor anonymity protects offspring from potential negative consequences for family relationships if the truth is revealed (19).

There are few publications comparing disclosing and nondisclosing families, and apart from some conflicts observed between mother and children among these families (20), parent-child relationships and children’s socioemotional functioning were found to be equal when children who had been told were compared with those who had not been told (20–24).

Although there is a rich body of literature on the discussion about anonymous or identified gamete donation, there is limited knowledge of patients’ attitudes towards disclosure issues, especially in countries where anonymous donation is required by law. It is also unknown whether the assisted reproduction treatment, and its physical and emotional implications could influence the opinion of individuals regarding donor anonymity.

Therefore, the goal of the present study was to investigate whether couples undergoing infertility treatments have a different perception of anonymous versus identity-release gamete donation than a target population in a country (Brazil) where anonymous donation is required by law.

## Methods

### Design:

This cross-sectional study was performed from September 1, 2020 to December 1, 2020, in which surveys were conducted through online platforms, including either patients undergoing ART (ART-group, n=400) or those interested in the subject who accessed the website of a university-affiliated IVF center (interested-group, n= 100). Participants were randomized by age, in a 1:4 ratio, into one of the two groups. Those in the ART group were invited via e-mail, with a cover letter outlining the survey and a link to access it, and participants in the interested-group obtained the questionnaire via a website. The survey collected information on demographic characteristics and participant attitudes towards anonymity of gamete donors, and answers were compared between the groups.

This study was approved by the Faculdade de Medicina de Jundiaí, Institutional Review Board, and participants provided the con-sent for publication of questionnaire data.

### Questionnaire:

The questionnaire contained questions regarding demographic data. Participants were asked to provide information about age (open response format), professional activity (open response format), and marital status (response options: marriage, single or common law relationship). There were three more questions regarding their perceptions concerning the anonymity of gamete donors:

i) In the case of children conceived through ART, do you believe that revealing the method of conception may affect the relationship between children and their parents?
  Response options: “Yes, it may affect the relationship with their parents” or “No, it wouldn’t affect the relationship with their parents”.
ii) In the case of oocyte donation, if the method of conception is revealed, do you believe that the child has the right to know the oocyte donor? Response options: “Yes, the child has the right to know his/her origin” or “No, the child should not be told”.
iii) If you had to reveal the identity of the gamete donor to your child, when do you think that the identity of the oocyte donor should be disclosed? Response options: “the oocyte donor’s identity should be revealed if the child questions its biological origin”, “the oocyte donor’s identity should be revealed since birth”, “the oocyte donor’s identity should be revealed when the child turns 18 years old”, or “the oocyte donor’s identity should be revealed sometime during teenage years”.
iv) In the case of sperm donation, if the method of conception is revealed, do you believe that the child has the right to know the sperm donor? Response options: “Yes, the child has the right to know his/her origin” or “No, the child should not be told”.
v) When do you think that the identity of the sperm donor should be disclosed?
  Response options: “the sperm donor’s identity should be revealed if the child questions its biological origin”, “the sperm donor’s identity should be revealed since birth”, “the sperm donor’s identity should be revealed when the child turns 18 years old”, or “the sperm donor’s identity should be revealed sometime during teenage years”.

### Statistical analysis:

Data were analyzed using the SPSS Statistics *vs*. 21 (IBM, USA) statistical program. Variables were tested for normality distribution and group homogeneity using the Shapiro Wilk and Levene’s tests, respectively. Age was compared between the groups using Student’s t-test, while other variables were compared using the Chi-square test. Age was described as the mean±standard deviation, and the other variables were described as the percentage±standard deviation. The considered significance level α was 5%.

## Results

There was no difference in age of two groups (38.3±7.2 *vs*. 36.9±6.3, p=0.076, for ART-group and interested-group, respectively, Table 1). Among ART-group, 86 cycles (21.5%) included 46 (11.5%) sperm donation and 40 (10%) egg donation. Most participants stated that the relationship between children and their parents would be affected by the child’s knowledge of the origin of its conception, regardless of the group (83.6% *vs*. 82.7%, for ART-group and interested-group, respectively, p=0.868, Table 1).

**Table 1.** Distribution of age and answers to questions

**Variable**	**ART-group 400**	**Interested-group 100**	**p-value**
**Age (years old)**	38.3±7.2	36.9±6.3	0.076
**Participants attitudes**			
The relationship between children and their parents would be affected by the child’s knowledge of the origin of its conception: answer YES (%)	83.6	82.7	0.868
The child has the right to know the oocyte donor: answer no (%)	65.4	50.8	0.044
The child has the right to know the sperm donor: answer no (%)	64.8	50.8	0.050

When asked if the sperm donor should be identifiable, most participants in the ART-group answered that the sperm donor identity should not be revealed to the child, while only half of the interested-group stated the same (65.4% *vs*. 50.8%, p=0.044, Table 1). The same result was observed when participants were asked if the oocyte donor should be identifiable (64.8% *vs*. 50.8%, p=0.050).

When asked when the donor’s identity should be revealed to the child, no significant differences were noted in the responses between the two groups. Most participants who believed that the child has the right to learn the donor’s identity stated that “the donor’s identity should be revealed if the child questions its biological origin” (67.2% *vs*. 67.5% for the ART-group and interested-group, respectively). “From birth” was the second most common response (21.0% *vs*. 19.7%, for ART-group and interested-group, respectively), while “when the child turns 18 years old” (9.2% *vs*. 11.2%, for ART-group and interested-group, respectively), and “sometime during teenage years” (2.5% *vs*. 2.4%, for ART-group and interested-group, respectively) were less common answers (Table 2).

**Table 2.** Opinions about when the donor’s identity should be revealed to the child in participants in ART-group and interested-group

**Opinions**		**ART-group 400**	**Interested-group 100**	**p-value**
When the donor’s identity should be revealed to the child	If the child questions its biological origin (%)	67.2	67.5	0.978
	From birth (%)	21.0	19.7	0.876
	When the child turns 18-years old (%)	9.2	11.2	0.865
	Sometime during teenage years (%)	2.5	2.4	0.789

## Discussion

There is a global trend towards open-identity gamete donation, with an increasing number of countries introducing legislation allowing only “open-identity” donors (9). However, identity-release donation may pose greater challenges to parents. For the present study, a survey was conducted through online platforms to investigate attitudes towards anonymous versus identity-release gamete donation in patients undergoing ART and a target population interested in ART in Brazil.

First, the participants were asked whether they believed that the relationship between children and parents would be affected by the child’s knowledge of the origin of its conception. Both participants in the ART- and interested-groups believed that the family relationship could be affected if the child was aware of the origin of his/her conception. In the case of gamete donation, there was a different perception among couples undergoing ART and those who accessed websites. Most intended parents believed that the identity of the gamete donor, sperm or oocyte, should not be revealed to the child, while only half of participants interested in ART stated the same.

When a couple conceives using donor oocytes or sperm, a family where the child has a genetic link to only one of the parents is created. It has been suggested that the absence of a genetic linkage as well as the existence of an identifiable donor may have psychosocial consequences for the couple and the family (7).

In countries where the legislation gives children, conceived through donation treatment, the right to obtain identifying information about the donor, parents of gamete donation children have no right to access identifying information about the donor but are encouraged to start disclosing the nature of the conception to the child from an early age (25). Despite this, previous studies have shown that disclosure of the gamete donor identity to the child is not an obvious decision for the parents (25–27).

The present study was performed in Brazil, where donor anonymity is mandatory by law. Although it has been suggested that parents using identifiable donors are more likely to disclose information (25, 28) in the UK, no significant increase in the rate of disclosure has been observed following the introduction of legislation mandating identifiable donation in 2005 (29). A review of the factors that might contribute to parents’ decision-making about disclosure showed that the impact of legislation on parents’ disclosure decisions is unclear (19).

The Brazilian law may have influenced participants’ answers, especially among couples undergoing ART. The reason that most of the participants undergoing ART believed that gamete donors should not be identifiable while only half of the interested-group stated the same was not elucidated; but it could be argued that the decision for disclosure may be more challenging when parenthood is a real and tangible issue. On the other hand, decisions may be different when one is only an observer of the situation. In previous reports, among parents who have used identifiable donors, the prospect of disclosure has been described to be associated with the fear that off-spring could form an attachment to the donor, fear of hurting the child, damaging the relation-ship or being rejected by the child, and among parents who have used anonymous donors, it has been argued that disclosure is unnecessary or may even be frustrating for offspring, who remains unable to access identifying information (28, 30–33).

The American Society for Reproductive Medicine (ASRM) argues for the right of the child to be informed about the origin of his/her conception and about the donation (34). In fact, according to the European Society of Human Reproduction and Embryology (ESHRE), there is no solution for the problem of donor anonymity. Many different rights are at stake including the right of autonomy and privacy of the parents, the right to privacy of the donor, and the right of the child to know his/her origins. Reports in favor of disclosure are in accordance with studies in the field of adoption, showing that children are not harmed by the truth about their genetic origin but by lack of such information. In fact, children benefit from an honest and positive relationship with their parents (25).

While donor anonymity was the rule in most countries in 1990s, identity-release donation is nowadays mandatory in at least 14 countries (9, 35). At the same time, professional opinion has moved towards acceptance of identity disclosure to children; as an example, ASRM guidelines changed from recommending anonymous donation in 1993 to accepting known donation in 2002 (36). A growing number of parents now disclose their origins to their donor-conceived children (33, 37).

In our study, among participants who stated that the children should be informed about their genetic origin and that the gamete donor should be identifiable, most were unsure about suitable timing for disclosure. One-third of them answered that the donor’s identity should be revealed if the child questions its biological origin. The second most common response was “since birth”, while “when the child turns 18 years old” and “sometime during teenage years” were less common answers.

When children who were conceived through gametes from identity-release donors grow up, parents need to deal with the question of disclosure to the child. In fact, in a previous report from Sweden, it was observed that as children grow older, reaching an agreement about what to tell the child concerning donor conception might be a source of stress in parenthood (38). It has previously been described that disclosure should not be seen as a single occasion but as a gradual process that may continue for years at different stages where parents talk to their children about how their family was formed rather than about how the children were conceived (39, 40).

It has been reported that a common age for disclosure to donor-conceived children is when they are approximately 5 years old (31, 33, 41). Children who have been told about their origin in the preschool years tend to cope well with their donor conception, with some of them reacting with curiosity and others showing no interest in the information (30, 42, 43). Indeed, it has been shown that those who knew about the donation from a young age felt neutral about it (44, 45), while those who learned about their genetic origin during adolescence or adulthood found it more traumatic and had difficulty coping with the issue (44–46).

The perception that the child is too young and needs to reach sufficient maturity to understand donor conception is a commonly reported reason to postpone disclosure (19, 47). In fact, postponing disclosure until the ‘right-time’ has been found to be risky, as the proposed perfect moment for disclosure may never appear (48), and sharing this information has been suggested to be more difficult the longer the parents wait (49). Irrespective of the age of the child at disclosure, most parents felt relieved after disclosure and did not regret the disclosure decision (30, 31, 43).

Lack of adequate opportunities to conduct face-to-face interviews and lack of knowledge of the real condition of the website participants concerning infertility or being involved in ART may have biased these results. While there is a movement in favor of a more open approach regarding disclosure of donor identity which is inspired by the United Nations Convention on the Rights of the Child (50) in many countries, the Brazilian legislation goes against this movement and it may influence the population opinion. The practice of donor-assisted conception is growing, and guidance is crucial to help these recipients/intended parents and offspring pass through the changing environment in which donor-assisted conception takes place.

## Conclusion

Despite the global tendency towards identity-release gamete donation programs, donor anonymity is still mandatory in Brazil, and attitudes among ART patients seem to be more conservative, with most participants believing that family relationships may be affected if the child is aware of the origin of his/her conception. In addition, these patients believe that the identity of the gamete donor should not be revealed to the child. Participants interested in ART seem to have an open-minded view; however, if facing the problem, future parents’ intentions to disclose may not be borne out in practice. Finally, despite the children’s right to know their biological origin, disclosure of the donor conception to offspring remains a challenge for many of parents.
